# The Lifespan Disparity Dataset: An open repository on inequality and polarization in length of life (1950–2021)

**DOI:** 10.1038/s41597-024-03426-6

**Published:** 2024-06-21

**Authors:** Vanesa Jorda, Miguel Niño-Zarazúa, Mercedes Tejería-Martínez

**Affiliations:** 1 Department of Economics, Universidad de Cantabria, Santander, 39300 Spain; 2 Department of Economics, SOAS University of London, London, WC1H 0XG UK; 3 United Nations University World Institute for Development Economics Research (UNU-WIDER), Helsinki, FI-00160 Finland

**Keywords:** Economics, Interdisciplinary studies

## Abstract

Monitoring health is key for identifying priorities in public health planning and improving healthcare services. Life expectancy has conventionally been regarded as a valuable indicator to compare the health status of different populations. However, this measure is simply the mean of the distribution of the length of life and, as such, neglects individual disparities in health outcomes. In this paper, we use life tables from the UN World Population Prospects to develop the most comprehensive dataset of lifespan inequality and polarization for 258 countries and areas for the period 1950–2021. These extensive series on lifespan distributions provide access to crucial information for researchers, practitioners, and the general public, thus contributing to a better understanding of health differences within and between nations.

## Background & Summary

Life expectancy is a commonly used health indicator that measures the average number of years a person is expected to live, based on various demographic factors including age, gender, and country of residence. This indicator is seen as a valuable tool for assessing the overall health and well-being of a population^1^. Life expectancy is used to monitor trends in health outcomes and to compare the health status of different populations^2,3^. For instance, countries with higher life expectancies generally have better access to healthcare and sanitary conditions.

Despite its popularity, life expectancy encounters several limitations as a health indicator. It does not take into account differences in the quality of life or health status among individuals within a population^4^. In this sense, a person may live a long life but experience poor health, disability, or limited mobility. Moreover, life expectancy can be influenced by factors such as socioeconomic status, lifestyle choices, and environmental factors, which can vary widely across individuals and cause differences in health outcomes. In addition, life expectancy does not capture health inequality^5,6^.

To address this last limitation, lifespan inequality could serve as an effective summary measure of mortality rate differences across age groups. Tracking the evolution of lifespan inequality is crucial because it helps promote social justice and equity by identifying and addressing the root causes of these disparities. Measuring lifespan inequality can also be used to compare health outcomes across different countries. This information is crucial for identifying areas where countries can learn from each other and collaborate to enhance global health standards. This is particularly important in driving global action toward achieving Sustainable Development Goal 3, which aims to ensure healthy lives for all ages.

To highlight the significance of this topic, it is important to underscore the extensive literature dedicated to exploring lifespan inequality. Table 1 presents a summary of the characteristics of the main body of research focusing on lifespan inequalities. Researchers have delved into this area from various perspectives, employing different inequality measures that include, among others, the Gini coefficient, Theil index, life disparity, variance, standard deviation, Heyfitz’s index, and interquartile range. As discussed in the Methods section, each measure offers unique insights into the distribution of lifespans within populations, shedding light on the magnitude and patterns of health inequalities.

**Table 1:** Inequality measures used by prior research across different regions.

	East Asia and the Pacific	Europe and Central Asia	Latin America and the Caribbean	North America	World	No real data used
Variance	^38,39^	^13,38,39^		^38,39^	^6,37,40^	
Standard deviation	^9,39,41^	^9,13,39,41–43^	^44^	^9,39,41^	^18,29,45,46^	
Interquartile range	^19^	^13,19^		^19^	^18^	
Life disparity	^47,48^	^13,20,21,43,48–50^		^50,51^	^5,35,52,53^	
Absolute Gini index		^43^			^52,54^	
Gini index	^9,55^	^8,9,13,42,43,55^		^8,9,55,56^	^6,14,22,35,54^	^57^
					^11,29,37,53^	
Keyfitz’s measure		^43,58^		^58^	^22,35^	
Theil index		^13,37,43,50^		^50^	^6,11,37^	
MLD		^13^				
Other measures	^9,41,48,55,59^	^9,36,41,55,60,61^		^9,36,41,55,62^	^18,22,35,45^	^57,63^
		^43,48^			^6,37,54,64–66^	

Closely related to the concept of inequality, polarization measures capture how concentrated the distribution is into different poles^7^. This phenomenon is of particular interest for the analysis of lifespan distributions. The shape of the distribution of length of life is typically characterized by two peaks due to the two underlying phenomena driving mortality patterns. The probability of dying decreases steadily until the age of 15, when the distribution becomes bell-shaped, representing adult mortality patterns^8–10^. The combination of high infant mortality rates and high adult mortality rates leads to a polarized distribution of lifespans, with a large number of deaths occurring at very young ages and at older ages. Public health practitioners can benefit from analysing the evolution of polarization in length of life as they can identify areas (infant or adult mortality) where health interventions are needed the most. By examining shifts in mortality patterns across different age groups, officials can detect segments of the population experiencing disproportionate mortality rates, whether in infancy or adulthood. Improving our understanding in this area could inform health policies that, for example, prioritize early mortality and social protection schemes aimed at protecting vulnerable individuals and groups^5^ Prior research has also underscored that efforts to decrease lifespan inequality may rely on policies that may not necessarily contribute to achieving progress in other health outcomes such as life expectancy^11^.

This paper contributes to the literature by developing a comprehensive set of estimates of length of life inequality and polarization for 258 countries/areas for the period 1950–2021. The full set of estimates is available at 10.6084/m9.figshare.24632181.v6^12^. We pay particular attention to additively decomposable inequality measures for practical purposes. Indeed, the use of this kind of measures makes it possible to compute lifespan inequality for any group of countries using only the information included in our dataset.

We disaggregate inequality patterns by sex due to the fact that lifespan distributions have been fairly distinct for men and women. Prior research has looked at adult and infant mortality separately because the factors that underline these two phenomena are aetiologically different^9–11^. Therefore, we include estimates of different inequality measures for both total population and the population aged over 15. Polarization measures are provided for the total population only. Given the shape of lifespan distributions, restricting the sample to the population aged over 15 results in a bell-shaped distribution, so polarization and inequality will both measure the same phenomenon.

A newly developed dataset containing different statistics on lifespan distribution is a significant contribution to the fields of demography and public health. This dataset has been created to measure the differences in lifespan across different populations and countries, providing valuable insights into the distribution of health and mortality between and within countries. This dataset will help inform policy decisions and resource allocation by highlighting the disparities in health outcomes within populations, and identify areas where interventions are needed to address health inequalities. Therefore, the dataset has the potential to contribute significantly to our understanding of global health disparities and to inform efforts to improve health equity.

## Methods

### Conceptual framework

As our dataset provides information about inequality and polarization in lifespans, an illustration of how to interpret these statistics is fundamental. In this section we explain how inequality and polarization measures are computed and how they relate to life expectancy, with a particular emphasis on the different phenomena that these two concepts capture. Life expectancy, inequality, and polarization in length of life are derived from period life tables. Abridged period life tables provide information on death rates for *K* = 24 age intervals: 0–1, 1–4, …, 5–9, 95–99, 100+. These figures can be seen as points of the probability density function (pdf) of the distribution of length of life, which gives the probability of dying in each interval. Life expectancy at birth, defined as the number of years that an individual born today might live if the current mortality patterns remained, is the mean of this distribution:

$$\mu =\mathop{\sum }\limits_{k=1}^{K}\,{f}_{k}\ast {c}_{k}$$

Where ${c}_{k}={x}_{k-1}+{a}_{k}$ is the average age at death in that interval, and ${f}_{k}={d}_{k}/{\ell }_{0}$. Both *a*_*k*_ and *d*_*k*_ are components of period life tables, where *d*_*k*_ represents the number of deaths in the interval ${I}_{k}=\left[{x}_{k-1},{x}_{k}\right]$ and *a*_*k*_ is the average time spent in the interval *I*_*k*_ by individuals who die before the age of *x*_*k*_. Finally, ${\ell }_{0}=1{0}^{5}$ is the size of the synthetic cohort.

Life expectancy is a powerful tool for tracking the evolution of health conditions in a country, as it summarizes mortality risks at various ages into a single figure. However, this measure does not capture differences in lifespan—or age at death—among individuals. Even if a country  shows progress on average, this does not guarantee health improvements for every member of society. Furthermore, two countries with the same life expectancy can exhibit significantly different patterns in the distribution of lifespans.

Inequality measures play a crucial role in understanding the disparities in lifespans among the citizens of a country. However, it is essential to recognize that the choice of measure can significantly influence the conclusions drawn about the direction of changes in inequality. The underlying sensitivities to different parts of the lifespan distribution vary across inequality measures^13^. Hence, depending on the specific measure used, not only the levels but also the trends in inequality may vary^14^.

Inequality indices can be classified into absolute or relative measures. To illustrate the distinction between these two types of measurements, consider the following example. Suppose we want to assess inequality between two individuals in two different countries. In Country A, one person lived for 6 years while the other lived for 60 years. In Country B, one individual lived for 7 years and the other lived for 70 years. Relative inequality measures would indicate equal levels of inequality in both countries because the distribution of lifespan in Country B can be derived from Country A by increasing the age at death by 17 per cent for both individuals. As a result, the relative difference between the two individuals in these two countries remains the same, at 1/10. On the other hand, absolute measures would deem Country B as more unequal, as the absolute difference in lifespan between the two citizens is 63 years, whereas in Country A it is 54 years. Hence, the choice between absolute and relative inequality measures can impact not only the magnitudes but also the trends of health inequality.

There is an ongoing debate regarding the more suitable approach for evaluating health inequalities^15^. Relative indicators have gained appeal when examining income variables^16^, whereas for variables with boundaries, such as lifespan, absolute changes are considered a better alternative for measuring health inequality^17^. As a result, demographers often favour absolute measures, with variance and standard deviation being the most commonly used statistics^9,10^. For data grouped into age intervals, as presented in life tables, the variance is given by

$${{\rm{\sigma }}}^{2}=\mathop{\sum }\limits_{k=1}^{K}{\left({c}_{k}-\mu \right)}^{2}{f}_{k},$$

where *μ* is life expectancy at birth. The standard deviation is calculated as the square root of the variance.

The interquartile range also serves as a widely employed indicator in prior research^13,18,19^. This absolute measure of dispersion is given by:

$$IQR={q}_{3}-{q}_{1},$$

where *q*_1_ and *q*_3_ stand for the first and the third cuartile, i.e. the age such that ${\sum }_{k}\,{f}_{k}=0.25$ and ${\sum }_{k}\,{f}_{k}=0.75$.

It should be noted that this inequality measure would be completely insensitive to changes in the distribution of deaths within *q*_1_ and *q*_3_ when the total death numbers before and after these ages do not change. This means that, for instance, if *q*_1_ = 15 years (25,000 individuals in the synthetic cohort die at ages under 15), the measure would remain unaltered whether 24,000 die at ages under 14 and 1,000 die at the age of 15 or if 1,000 die at ages under 14 and 24,000 die aged 15. Hence, this limitation underscores the need for complementary measures to fully capture changes in mortality patterns across different age groups.

Life disparity is also a widely used absolute indicator of lifespan variation^5,20,21^. Its popularity can be attributed to its intuitive interpretation as the average remaining life expectancy when death occurs, or life years lost due to death^5^. When the length of life distribution is highly dispersed, several people die aged below their expected age at death, which would contribute to many lost years to life disparity. In contrast, if survival is concentrated among the elderly, the gap between the actual age at death and the expected remaining years decreases, consequently reducing life disparity. Life disparity can be expressed as^22^:

$${e}^{\dagger }=\mathop{\sum }\limits_{x=0}^{K}\,{f}_{x}\left[{e}_{x}+\frac{{a}_{x}}{n}\left({e}_{x+1}-{e}_{x}\right)\right].$$

Life disparity is closely related to the Keyfitz’s H measure, conventionally known as the life table entropy^23^. This statistic is a relative inequality measure which has an appealing interpretation as the elasticity of life expectancy to a proportional change in mortality. It informs about the percentage change in life expectancy as a result of a one percent reduction in the force of mortality at all ages^24^. The Keyfitz’s H measure can be defined as the ratio given by life disparity and life expectancy at birth^24,25^:

$$H=\frac{{e}^{\dagger }}{\mu }.$$

Among the relative measures, the popularity of the Gini index has spread to health variables^10,11,26^. Consequently, we also compute estimates for this inequality measure, which can be calculated as follows:

$$G=\frac{1}{2\mu }\mathop{\sum }\limits_{k=1}^{K}\mathop{\sum }\limits_{j=1}^{K}| {c}_{k}-{c}_{j}| \,{f}_{k}{f}_{j}.$$

The absolute version of this indicator is given by:

$$AG=\mu \ast G.$$

Despite the widespread acceptance of the Gini index, this measure has certain limitations. The index is sensitive to the middle of the distribution, but it does not allow for varying the weight assigned to differences in specific segments of the distribution. Inequality measures can capture diverse trends, depending on their sensitivity to different parts of the distribution. To address this concern, we calculate an alternative set of inequality measures from the generalized entropy (GE) family. This family includes a sensitivity parameter (*θ*) that enables us to adjust the importance attributed to disparities in lifespan among older individuals. The mean log deviation (MLD) corresponds to the GE index when the parameter is set to 0, which is more sensitive to infant mortality. The Theil’s entropy measure, characterized by a parameter value equal to 1, is equally sensitive to all segments of the distribution. We also compute the GE measure with *θ* set to 2 to examine the evolution of lifespan inequality with greater emphasis on differences among the elderly. The GE class of inequality measures is given by,

$$G{E}^{(\theta )}=\frac{1}{\theta (\theta -1)}\left(\mathop{\sum }\limits_{k=1}^{K}\,{f}_{k}{\left(\frac{{c}_{k}}{\mu }\right)}^{\theta }-1\right),\theta \ne 0,1.$$

where, for *θ* = 1, we have the following limiting case:

$$T=\mathop{\sum }\limits_{k=1}^{K}\,{f}_{k}\frac{{c}_{k}}{\mu }\log \left(\frac{{c}_{k}}{\mu }\right)$$

and for *θ* = 0, the index tends to

$$MLD=\mathop{\sum }\limits_{k=1}^{K}\,{f}_{k}\log \left(\frac{\mu }{{c}_{k}}\right).$$

While inequality measures can provide valuable insights about the level and evolution of differences in length of life, some aspects of the distribution can only be analysed with polarization indices. The concept of polarization relies on the alienation–identification framework^27^. This framework classifies individuals into groups (or poles). Individuals feel that they identify with other individuals in their group, while feeling alienated from those belonging to other groups. Polarization is a measure that summarizes the distance between these poles (alienation) and their representativity (identification). In the same vein as inequality, as the distance between these groups increases, the distribution becomes more spread and individuals feel more alienated from each other, also fostering polarization. By contrast, as individuals within a group become more similar to each other, particularly in terms of lifespans in the context of this study, their identification with each other grows stronger, which increases polarization. Identification is measured by the concentration of the probability mass around a pole.

The identification–alienation framework can be implemented using the following polarization measure^28^:

$$DER(\alpha )=\mathop{\sum }\limits_{k=1}^{K}\mathop{\sum }\limits_{j=1}^{K}\left|{c}_{k}-{c}_{j}\right|{f}_{k}^{1+\alpha }{f}_{j},$$

where *α* ∈ [0.25, 1] is the so-called ‘polarization aversion’ parameter, which captures the power of the identification effect. The greater the value of *α*, the greater the distinction between polarization and inequality. Indeed, when *α* = 0, this measure is equivalent to the absolute Gini index. Although the DER index measures absolute polarization, it can be easily transformed into a relative index by multiplying the DER index by *μ*^*α*−1^, where *μ* is the life expectancy.

While the identification–alienation framework was initially designed for income distributions, it can be applied to length of life distributions. As mentioned above, life tables are a statistical construct that do not reflect the mortality distribution of a real cohort, but rather the current mortality patterns in a given population. While individuals are mostly unaware of life table statistics, their knowledge about the survival chances of friends, relatives, acquaintances and other social groups seems to influence perceptions of survival expectations^29^. Previous research suggests that these subjective survival expectations can predict actual mortality rates and reflect established socioeconomic disparities in death rates^9^. The ability of the current cohort to observe changes in the distribution of length of life and react to them is precisely what makes the identification-alienation framework suitable for our analysis.

**Fig. 1:**
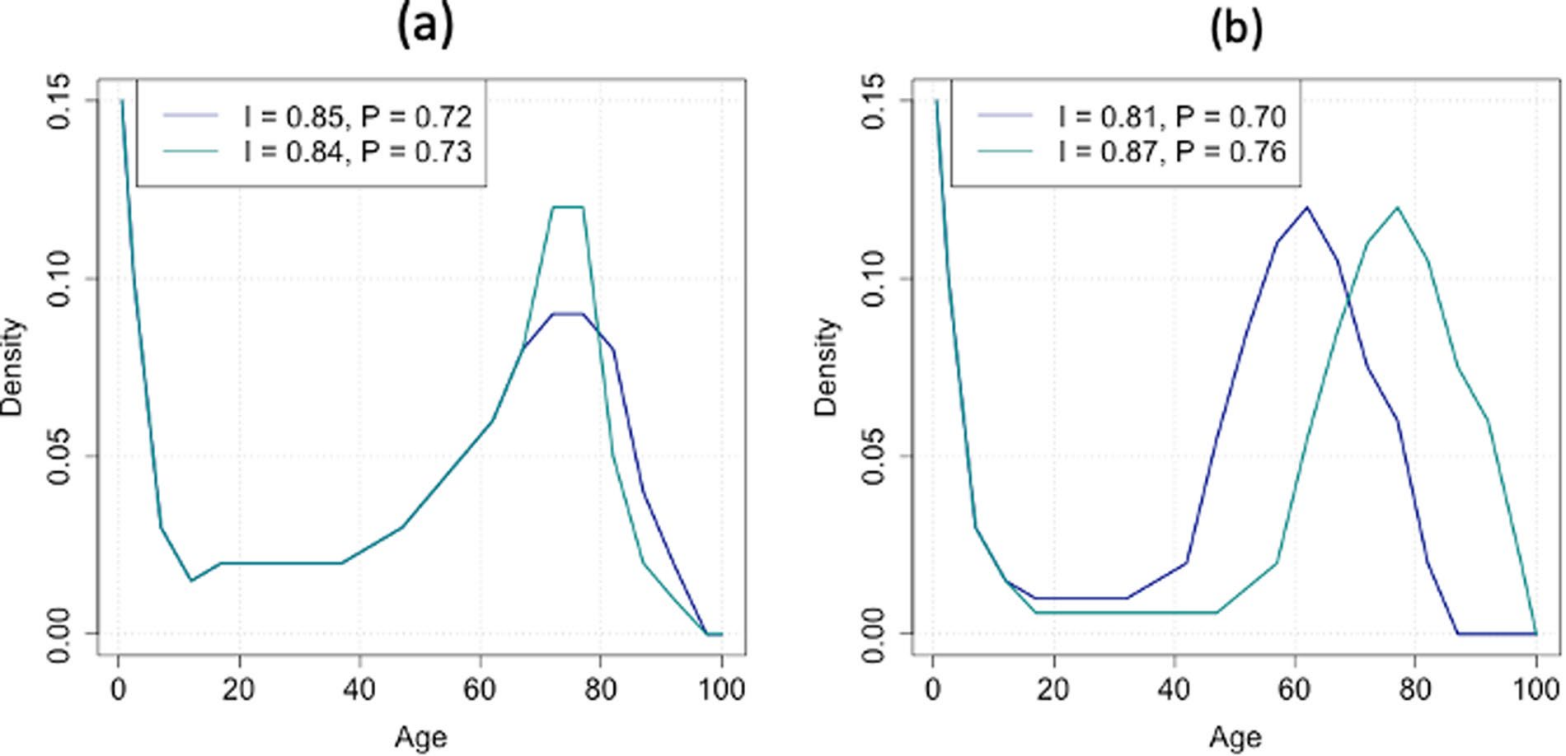
Changes in length of life distributions and its impact on inequality and polarization.

To illustrate this point, consider the scenario depicted in Fig. 1(a), which presents two lifespan distributions, identical up to the age of 65. These distributions show a first spike at the lower end, which depicts infant mortality patterns. The probability of dying decreases steadily until the age of 15, when the distribution becomes bell-shaped, with a second mode at 75 years. Assume that the transition from the blue distribution to the green distribution reflects a change in public health policy aimed at reducing premature deaths. As a result of this policy, the second mode of the distribution becomes more concentrated around modal age of death.

While this strategy reduces overall inequality in lifespan, it also leads to increased polarization. The MLD falls from 0.85 to 0.84, while polarization increases from 0.72 to 0.73. This hypothetical change in the allocation of healthcare spending in favor of initiatives to prevent premature mortality at the expense of reducing medical treatments for the elderly may cause discontent and social unrest, especially in contexts of ageing populations. The impact of such protests would be intricately linked to the representativity of this second pole (identification), comprised of individuals who experience heightened mortality rates due to the reallocation of healthcare resources. In other words, the extent of social discontent may be influenced by the magnitude of the population affected by reduced access to medical treatments, particularly among the elderly.

As for the alienation feeling, consider the scenario presented in Fig. 1(b). This population has made great progress towards reducing adult mortality, indicating advancements in healthcare and living conditions for the working-age population. However, simultaneously, infant mortality rates show no signs of reduction, indicating persistent challenges in maternal and child health care. In such a scenario, segments of the population, particularly parents and caregivers of young children, may experience a sense of alienation and discontent. This disparity in mortality outcomes between different age groups could exacerbate social tensions within society. As a result, polarization increases, and due to the increase in the spread of the distribution, inequality also rises.

### Data collection

To evaluate the level of inequality in length of life of a particular country in a given year, we need information about the death rate broken down by age and sex. Period life tables contain data on the number of deaths for every five-year age group up to 100 years for a synthetic cohort of 100,000 individuals. For a certain year, period life tables are constructed using data on the number of deaths in that particular year. Hence, mortality rates do not refer to the actual mortality patterns of a real birth cohort over its lifetime, but to the current mortality patterns of a country. Due to the large decreases in mortality rates over time–as a result of, for instance, medical advances–a person born today does not face the same probability of dying as a person born in 1900. Since we are interested in providing a snapshot of lifespan inequality trends, we use period life tables to perform the analysis as they provide an indication of the mortality situation at a particular point in time.

The data have been retrieved from World Population Prospects: The 2022 Revision, developed by the UN Population Division^30^. Among all the available sources, this dataset is the most appealing because of its geographical and temporal coverage. Detailed demographic estimates and projections on fertility, mortality, and migration are provided for every member state of the UN from 1950 onward. Hence, this data source provides a balanced panel of 237 countries/areas from 1950 to 2021 on an annual basis. Despite the wide geographic and temporal coverage of the data, the quality and consistency of mortality series vary substantially across countries. Estimates of mortality rates are derived directly from registered deaths when civil registration data of good quality is available. Although most countries presented vital registration coverage greater than 90 per cent in recent years, the period before 1990 was plagued by the lack of empirical data. When official demographic statistics are not reported in the detail necessary for the preparation of cohort population projections, the UN Population Division undertakes its estimation by using data from different sources, including major surveys such as the Demographic and Health Surveys or the Multiple-Indicator Cluster Surveys, population censuses, and analytical reports.

## Data Records

The dataset, available at figshare^12^, contains six files with information on national and regional estimates of inequality and polarization in length of life for 258 countries/areas for each year from 1950 to 2021. The datafile *MAY2024_both_total* includes the estimates for the whole population, whereas *MAY2024_both_15* gathers the data for the population aged over 15. Female estimates are stored in *MAY2024_female_total* and *MAY2024_female_15* for all women and those aged over 15 respectively. Finally, *MAY2024_male_total* includes the estimates for the male population, and *MAY2024_male_15* only for men aged over-15. The descriptions of the variables of these data files are presented in Table 2.

**Table 2:** Variable description.

Record	Description
country	Country name
year	Year
desa_id	Standard country or area codes for statistical use of the United Nations Statistics Division
country_year_id	Country–year identification code
ISO3	ISO country code three-digit
area_type_name	The type of area: country, world
SDG_reg_name	Geographic regions defined by the United Nations Statistics Division: Australia/New Zealand, Central and Southern Asia, Eastern and South-Eastern Asia, Europe and Northern America, Latin America and the Caribbean, Northern Africa and Western Asia, Oceania (excluding Australia and New Zealand), Sub-Saharan Africa
WB_inc_name	Income groups defined by the World Bank: high-income countries, low-income countries, lower-middle-income countries, and upper-middle-income countries
WB_reg_name	Geographic regions defined by the World Bank: East Asia and the Pacific, Europe and Central Asia, Latin America and the Caribbean, Middle East and North Africa, North America, South Asia, Sub-Saharan Africa
le	Life expectancy
theil	Theil index
mld	Mean log deviation
ge2	Generalized entropy measure (*α* = 2)
variance	Variance
stdev	Standard deviation
gini	Gini index
abs_gini	Absolute Gini index
iqr	Interquartile range
H_keyfitz	Keyfitz’s measure
life_disparity	Life disparity
ader_25	Absolute polarization measure (*α* = 0.25)
ader_50	Absolute polarization measure (*α* = 0.5)
ader_75	Absolute polarization measure (*α* = 0.75)
ader_1	Absolute polarization measure (*α* = 1)
rder_25	Relative polarization measure (*α* = 0.25)
rder_50	Relative polarization measure (*α* = 0.5)
rder_75	Relative polarization measure (*α* = 0.75)
rder_1	Relative polarization measure (*α* = 1)
adult_pop	Adult population
total_pop	Total population
source	Coverage and type of data used to construct the estimates

The file code *quality_data* gathers information on the type of data used to build our estimates for each country and year, retrieved from past editions of the Demographic Year Book. National estimates are classified into three broad categories: ‘C’ is used when civil data are expected to be virtually complete, representing at least 90 per cent of deaths; ‘U’ is for countries where vital information is expected to cover less than 90 per cent of deaths; and ‘I’ indicates that the data are not gathered from civil registration systems but are considered reliable. Countries classified within this last category present demographic information derived from projections, population and housing censuses, or other estimation techniques. Finally, ‘N’ is used for countries with no information about the completeness of the data. In some cases, deaths are tabulated by date of registration instead of by date of occurrence, indicated by the symbol ‘*’. This might affect mortality estimates if the lag between the date of occurrence and the date of registration is considerable, which would lead to a substantial delay in death registrations. However, delays in the registration of deaths are less common and shorter than those in the registration of live births.

## Technical Validation

**Fig. 2:**
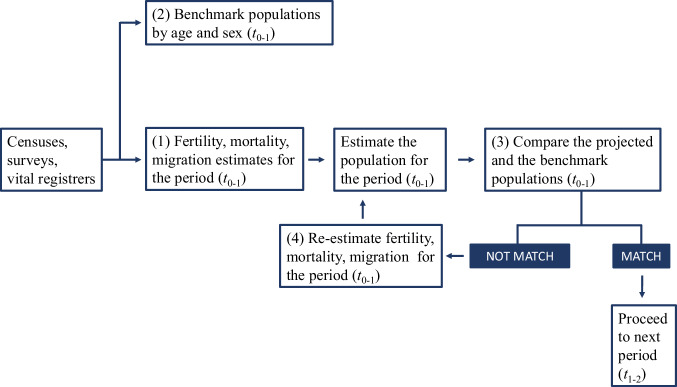
Procedure implemented in the estimation of UN World Population Prospects to ensure consistency between demographic components and population projections. Source: adapted from DESA^31^.

The reliability of our estimates crucially depends on the quality of the data sources used for the construction of the period life tables. UN-DESA relies on both civil and survey data to construct mortality estimates. The precision of these estimates is evaluated to identify the most likely series of mortality. The core methodology for the estimation of population estimates from the 2022 Revision of World Population Prospects is the cohort-component method for projecting population^31^. This approach for producing population estimates and projections involves several steps (Fig. 2).

1. The first step is to estimate the components of demographic change (fertility, mortality, and migration) using data from various sources, including censuses, surveys, vital and population registers, and analytical reports. Estimates from different sources might produce substantially different figures, which are consolidated through expert-based opinion or using automated statistical methods that generate a smooth trend. We refer the reader to https://esa.un.org/unpd/wpp/DataSources/ for a detailed description of the sources and DESA^31^ for the different procedures applied to construct the estimates of adult and child mortality.
2. The second step is to estimate benchmark populations by age and sex, using population counts from censuses and other sources. These counts are adjusted as necessary, and supplemental benchmarks are considered for specific age groups.
3. In the third step, the population for period *t* + 1 is computed from the population at period *t*, considering the births minus the deaths registered in that period and the net international migration. These figures are then compared with the benchmark population estimates from step 2 to assess their internal consistency. Another important aspect of the work at this stage is ensuring that information on the net number of international migrants is consistent and sums to zero at the world level.
4. The fourth step involves re-estimating the demographic components as necessary to achieve consistency between the estimated and the benchmark populations. A final consistency check is performed to ensure the geographical coverage and the demographic plausibility of these estimates are evaluated and, if necessary, adjusted to be congruent over time and across age groups.

This procedure has been deployed in each revision of the World Population Prospects. When new information becomes available, previous estimates are re-evaluated, which means that with every new revision demographic trends may be adjusted.

Although the methodological framework described above requires consistency with the estimated trends of fertility, mortality, and net international migration, our focus resides in mortality series as these data are the primary source to produce our estimates. The techniques employed to estimate mortality rates and life tables differ among countries, depending on the nature and reliability of the available data sources. These techniques can be broadly classified into two main types: *empirical* and *model-based*. The empirical method was deployed for those countries with accurate data that enabled the construction of sex- and age-specific mortality rates through vital registration or other reliable estimates across a large number of years for the period 1950–2021.

For those countries that lacked comprehensive information to use the empirical method, the model-based approach is deployed. A minimum of one parameter is required describing the mortality rate among children or overall (such as the life expectancy at birth). An extra parameter that accounts for adult mortality can be beneficial to select the most appropriate model to accurately represent the mortality age pattern for a specific country and year. To provide the necessary information to reconstruct period life tables, annual time series of complete child and adult mortality rates are estimated for each country from 1950 to 2021.

The core approach for estimating mortality is not appropriate for countries with a high prevalence of HIV and AIDS. As these diseases occur primarily among adults of reproductive age, they modify the conventional U-shaped age profile of mortality. In these countries, the method applied by World Population Prospects 2022 applies adjustments to model-based estimates because model life tables are not able to represent this atypical mortality pattern by age. A similar approach is applied to account for the excess of mortality due to conflicts, natural disasters, or epidemics.

Since the extent and the quality of the data used to construct mortality series is crucial to assess the accuracy of the estimates on lifespan inequality and polarization, our set of estimates includes information about the source of the data used by the UN World Population Prospects. This information is available in the file *quality_data* for each country/territory on an annual basis. A summary of this information is presented in Table 3, which presents the proportion of countries/territories whose mortality series are constructed from reliable estimates or civil data that can be complete, incomplete, or unknown, for infant and adult mortality.

**Table 3:** Data coverage of the sources used to construct period life tables.

	Both complete	Both incomplete	Both unknown	Reliable estimates	Complete/ incomplete	Complete/ unknown	Incomplete/ unknown
1950	19.92	6.36	63.98	0	0.42	1.69	7.63
1970	40.25	17.37	34.32	0.42	1.69	1.69	4.24
1990	44.07	7.63	40.25	0	1.69	1.69	4.66
2010	52.97	11.86	23.31	5.08	0.42	2.54	3.81
2021	50	10.59	27.12	2.12	0.85	5.08	4.24
EAP	34.58	16.7	43.17	0.69	0.15	1.24	3.47
ECA	84.48	0.8	13.38	0.83	0.15	0.37	0
LAC	48.88	26.09	18.12	0.3	1.03	3.8	1.79
MENA	21.04	25.07	35.56	0.76	1.25	1.81	14.51
NA	94.91	0	0.46	0	2.78	1.85	0
SA	13.19	16.84	60.07	5.21	0	3.47	1.22
SSA	10.19	6.66	74.97	1.56	1.01	0.64	4.98

World Bank regions: EAP, East Asia and the Pacific; ECA, Europe and Central Asia; LAC, Latin America and the Caribbean; MENA, Middle East and North Africa; NA, North America; SA, South Asia; SSA, Sub-Saharan Africa.

Source: authors’ calculations from Demographic Year Books 1950–2021.

There has been a significant improvement in data coverage over time. In 1950, a substantial portion of life tables relied on data whose origin and quality were unknown (63.98 per cent). However, by 2021 this fell to 30.51 per cent, reflecting the wider availability and the improved reliability of data sources. The proportion of countries with civil data that cover more than 90 per cent of the adult and infant populations (labelled as *both complete*) also increased over the years, which reflects more comprehensive coverage of life table data.

Despite the advances in data collection methodologies and the efforts made to enhance the quality of demographic data, Table 3 highlights regional variations in data coverage. For instance, North America and Europe and Central Asia exhibit the highest percentage of data classified as *both complete* at 85 and 94 per cent, respectively. These figures indicate that these regions include countries with robust data collection systems. By contrast, only 10 per cent of South Asian estimates are based on complete civil data. This points towards a need for further efforts to improve data collection and quality in this region. Similarly, in the Middle East and North Africa a large proportion of life tables are constructed using incomplete or unknown data, which reveals important challenges in obtaining complete and reliable information in these countries.

Even though some data issues remain and the procedures implemented to obtain the estimates rely, in most cases, on statistical assumptions, the UN Population Division has managed to produce internally consistent and plausible estimates on fertility, mortality, and migration. Life expectancy and other life statistics provided by the World Health Organization are computed from the data provided by the UN World Population Prospects. Hence, even though we should be cautious using these series, we should also acknowledge that these figures are widely accepted and used to make cross-country comparisons, as these data satisfy the highest quality standards.

To minimize the risk of human errors in the development of this dataset, we used pair programming and thorough code reviews conducted by multiple team members to identify and rectify any potential issues. The estimation function of each indicator was developed in parallel by two members of our team. This parallel development approach served as an effective means of quality assurance, as any discrepancies between the results produced by these parallel implementations were flagged as potential errors. Upon completion of the parallel development phase, the results generated by the two sets of functions were compared. Any inconsistencies were scrutinized, and the source of the discrepancy was rectified. Once both sets of functions produced consistent results, we evaluated their computational efficiency. The implementation that demonstrated superior efficiency was ultimately integrated into the final script, ensuring not only accuracy but also optimized performance.

In addition to the aforementioned measures, we further bolstered the credibility of our results through manual verification. To validate the accuracy of our estimations, a subset of county-year observations was randomly selected. For each of these observations, inequality and polarization estimates were manually computed using established methodologies. This manual verification step provided an additional layer of assurance regarding the robustness of our methodology. Any discrepancies between the automated and manual computations were thoroughly investigated, allowing us to identify and rectify potential errors.

**Fig. 3:**
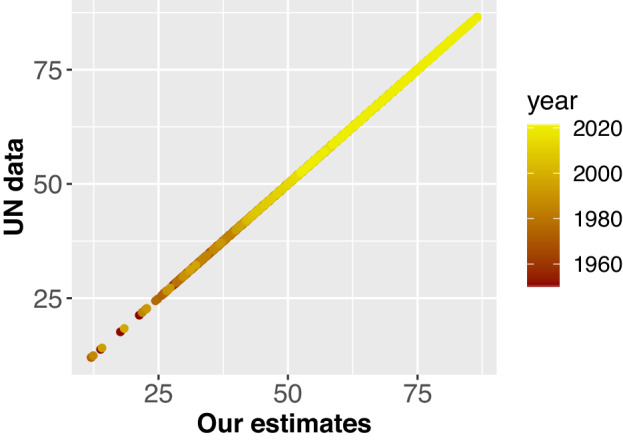
Association between our estimates and UN data on life expectancy.

As a final exercise to validate the reliability of our estimates, we compare official data on life expectancy released by World Population Prospects 2022 with our estimates. Since both estimates of life expectancy are derived from UN life tables, we anticipate that our results will closely align with the life expectancy series officially reported by the UN. Figure 3 plots our life expectancy estimates against UN figures, revealing a straight line, which indicates a strong relationship between them. In fact, the correlation coefficient is 0.999998, which confirms the existence of an almost perfect positive correlation between our estimates and the UN series.

## Usage Notes

The dataset on lifespan inequality and polarization contributes to analysing health trends, understanding disease burdens, and determining priorities for public health planning. Monitoring health inequality might also reveal differences in health outcomes within the population. This information is crucial for targeting interventions and policies aimed at reducing health disparities and promoting health equity. Moreover, these data are essential for the evaluation of the effectiveness and impact of health policies, interventions, and programmes. Tracking health indicators over time allows for evidence-based decision-making, and helps policy-makers to identify successful strategies and areas that require improvement^32^.

As our dataset covers virtually all countries, it enables international comparisons, which helps identify successful health policies and interventions implemented in countries with better health standards^33,34^. Its wide geographical coverage can also contribute to expanding the literature on the relationship between life expectancy and life disparity, as well as the underlying causes^22,35,36^. By encompassing diverse populations across the globe, our dataset offers a rich resource for researchers and health practitioners to explore regional and global trends in lifespan inequality and polarization^6,8,20,37^.

To help with the visualization and the use of these estimates, we have designed a user-friendly website available at https://vanesajorda.shinyapps.io/App-lifespans/. This tool has been designed to enable users to view, plot, and download national and regional estimates and lifespan inequalities for any group of countries of their choice, thus providing access to crucial information for researchers, policy-makers, and the general public.

## Data Availability

The source code to compute the dataset and reproduce the figures presented in this paper is available on Github repository https://github.com/vjg65/Length-of-life-inequality-and-polarization.
